# Application of the maximum threshold distances to reduce gene flow frequency in the coexistence between genetically modified (GM) and non‐GM maize

**DOI:** 10.1111/eva.13361

**Published:** 2022-03-11

**Authors:** Ning Hu, Ji‐chao Hu, Xiao‐dong Jiang, Wei Xiao, Ke‐min Yao, Liang Li, Xin‐hai Li, Xin‐wu Pei

**Affiliations:** ^1^ Yale‐NUIST Center on Atmospheric Environment International Joint Laboratory on Climate and Environment Change Nanjing University of Information Science & Technology Nanjing China; ^2^ Jiangsu Key Laboratory of Agricultural Meteorology Nanjing University of Information Science & Technology Nanjing Jiangsu China; ^3^ 12661 Biotechnology Research Institute Chinese Academy of Agricultural Sciences Beijing China

**Keywords:** gene flow, genetic competitiveness, maize, maximum threshold distance (MTD), risk management

## Abstract

On the coexistence of genetically modified (GM) and non‐GM maize, the isolation distance plays an important role in controlling the transgenic flow. In this study, maize gene flow model was used to quantify the MTD_0.1%_ and MTD_1%_ in the main maize‐planting regions of China; those were the maximum threshold distance for the gene flow frequency equal to or lower than 1% and 0.1%. The model showed that the extreme MTD_1%_ and MTD_0.1%_ were 187 and 548 m, respectively. The regions of northern China and the coastal plain, including Hainan crop winter‐season multiplication base, showed a significantly high risk for maize gene flow, while the west‐south of China was the largest low‐risk areas. Except for a few sites, the isolation distance of 500 m could yield a seed purity of better than 0.1% and meet the production needs of breeder seeds. The parameters of genetic competitiveness (*cp*) were introduced to assess the effects of hybrid compatibility between the donor and recipient. The results showed that hybrid incompatibility could minimize the risk. When *cp* = 0.05, MTD_1%_ and MTD_0.1%_ could be greatly reduced within 19 m and 75 m. These data were helpful to provide scientific data to set the isolation distance between GM and non‐GM maize and select the right place to produce the hybrid maize seeds.

## 1 INTRODUCTION

Maize was one of the first genetically modified (GM) crops and has been used commercially since 1996. In 2019, the global planting area of transgenic maize was 60.9 million hectares making it the second‐largest GM crop after soybean (ISAAA, 2021). In China, we already obtained a batch of GM maize materials with independent intellectual property rights. Following the 2009 approval of phytase maize, the Ministry of Agriculture and Rural Affairs of China approved the production and application of insect‐resistant and herbicide‐tolerant GM maize “DBN9936,” “Ruifeng125,” “DBN9501,” and herbicide‐tolerant GM maize “DBN9858” in January 2021 (MARA, 2021). These are important initiatives to protect the maize seed industry and stabilize maize production in China. Gene flow from GM crops to the same species or wild relatives is a major concern in risk assessment. Strict gene flow tests must be carried out before transgenic crops can be commercialized. Results submitted to regulators include gene flow frequency, gene flow distance, effects on related wild species, and potential for weediness. Therefore, in the commercial application of GM maize, various countries attach great importance to the risk prevention and management of the coexistence of GM and non‐GM maize.

Maize has a high natural outcrossing rate, and its pollen can survive for a long time before fertilization, both of which increase the gene flow risk at the longer distances. Temporal separation and distance isolation are, therefore, necessary to control these risks when the coexistence of genetically modified (GM) and non‐GM crops (Devos et al., 2005). A time lag of flowering synchrony between the neighboring maize could significantly reduce the extent of cross‐fertilization effects (Devos et al., 2014). The results demonstrated that there were no obvious differences in gene flow frequency when the flowering phase was separated by three days; however, when the flowering phases were within four–five days and six days of temporal separation, the gene flow frequency reduced by 25% and 50%, respectively. When the flowering phases were more than seven days apart, the gene flow frequency was less than 0.9%, even for adjacent planting (Bannert et al., 2008; Della Porta et al., 2008). Although temporal isolation used less land, the flowering phases varied with temperature, light, moisture, and nutrient conditions. This made it difficult to adjust the flowering phase, especially in cold northern regions with limited thermal resources. In contrast, distance isolation was simpler and equally effective. It was demonstrated that isolation distances of 150 m could reduce the gene flow frequency to below 0.1% (Ao et al., 2011). However, different countries and institutions have different regulations on isolation distances. In EU, the mandatory isolation distances between GM maize and non‐GM maize are 15–800 m, and the distances required for the organic maize are greater far; the organic farms must separate 250–800 m from GM maize in Denmark, Hungary, Luxembourg, Netherlands, and Spain (Devos et al., 2009; Riesgo et al., 2010; Sanvido et al., 2008). China has not yet regulated isolation distances for GM maize and non‐GM maize, but a reference isolate distance of 300 m was proposed in the supporting policies of agricultural GMO safety supervision (MARA, 2022). However, in practice it was very difficult to implement isolation distance of 300 m.

The data from field experiments, especially the distances where the gene flow frequency is less than a certain threshold value of 1% or 0.1% (MTD_1%_ or MTD_0.1%_), could provide important reference to set a suitable isolation distance for transgenic maize (Ao et al., 2011). However, many factors affect gene flow in maize, the most important of which is the local climate and cross‐fertilization (Devos et al., 2009). It was shown that MTD_1%_ and MTD_0.1%_ obtained in various regions could differ by more than 10 times (Table 1); therefore, it must be noted that experimental data were locally limited. As the most important food crops in China, maize is widely distributed across China from Hainan island in the south (18° N latitude) to Heihe River in the north of Heilongjiang Province (53°N latitude). However, there is a lack of systematic research and scientific data on maize gene flow. Whether the gene flow data and isolation distance from other countries could be applied to China's climate condition need to be studied further.

**TABLE 1:** Summary of the MTD_1%_ and MTD_0.1%_ of maize in the field experiments

No.	Site	MTD_1%_	MTD_0.1%_	Reference
1	Heilongjiang, China	60	112	Di & Liu (2008)
2	Shandong, China	60	119	Lu et al. (2005)
3	Shandong, China	45	200	Liu et al. (2015)
4	Jilin, China	20	‐	Lu et al. (2012)
5	Hainan, China	15	40	Zhang et al. (2020)
6	Chiayi, Taiwan	50	‐	Wang et al. (2013)
7	Wufeng, Taiwan	2.25	‐	Nieh et al. (2014)
8	Hokkaido, Japan	70	780	Kawashima et al. (2011)
9	Gunma, Japan	36	‐	Ushiyama et al. (2009)
10	Groß Lüsewitz, Sickte and Rheinstetten‐Forchheim, Germany	5	‐	Rühl et al. (2011)
11	Mariensee, Wendhausen, Braunschweig and Dahnsdorf, Germany	60	‐	Langhof et al. (2008)
12	Bavaria, Brandenburg, Baden‐Wurttemberg, Mecklenburg‐Pomerania, Saxony, Saxony‐Anhalt and Thuringia, Germany	60	‐	Weber et al. (2007)
13	Wendhausen and Groß Lüsewitz, Germany	102	‐	Langhof et al. (2008); Langhof et al. (2010)
14	Po Valley, Italy	25	‐	Della Porta et al. (2008)
15	Drenthe, Flevoland, Noord Brabant, Limburg, Gronmgen, Gelderland and Zeeland, Netherlands	12	‐	Van De Wiel et al. (2009)
16	Catalonia, Spain	3	‐	Melé et al. (2015)
17	Girona, Spain	40	‐	Palaudelmàs et al. (2012)
18	Catalunya, Spain	32	71	Messeguer et al. (2006)
19	Girona, Spain	40	‐	Pla et al. (2006)
20	Mallorca, Spain	30	‐	VivesVallés et al. (2015)
21	Uri, Switzerland	‐	52	Bannert and Stamp (2007)
22	Zurich, Switzerland	6	‐	Bannert et al. (2008)
23	England and Scotland, UK	5	81	Weekes et al. (2007)
24	Iowa, USA	100	150	Goggi et al. (2006); Goggi et al. (2007)
25	California and Washington, USA	32	123	Halsey et al. (2005)
26	Maine, USA	>110	‐	Jemison & Vayda (2001)
27	Colorado, USA	46	183	Byrne & Fromherz (2003)
28	Nebraska, USA	3	25	Barnes et al. (2020)
29	Ontario, Canada	28	‐	Ma et al. (2004)
30	Minas Gerais and Sao Paulo, Brazil	100	‐	Nascimento et al. (2012)
31	Sinaloa, Baja California Sur, Sonora, Chihuahua, Coahuila and Tamaulipas, Mexico	20	‐	Baltazar et al. (2015)
32	Nayarit, Mexico	25	100	Luna et al. (2001)
33	Free State, South Africa	40	141	Viljoen & Chetty (2011)
Median value		32	116	

Note

Represents no data.

Previous studies have demonstrated that despite the larger and heavier of the maize pollen grains, they could still escape to at least 100 m from the pollen source (Boehm et al., 2008; Hofmann et al., 2010, 2014). When the length of pollen source was less than 100 m at the prevailing wind direction, the larger the pollen source, the more pollen grains deposited on the downwind and the greater gene flow risk. The experiment results also proved that the gene flow frequency would increase as the pollen source area enlarging (Lu et al., 2019; Palaudelmàs et al., 2012). However, the field experiments were so small, typically less than 1000 m^2^ (Di & Liu, 2008; Lu et al., 2005, 2012) in China, so as to underestimate the gene flow distances and not to provide accurate and reliable data for assessing the risk of maize gene flow. The pollen source area more than 100 × 100 m^2^ made the field experiment harder, which was the main reason to use a maize gene flow model as the effective alternative for assessing the gene flow risk.

In conclusion, we identified 24 provinces, municipalities, and autonomous regions, including Heilongjiang, Jilin, and Liaoning (Table 2), which account for more than 98% of the total maize‐planting area in all of China (NBS, 2020). Using our maize gene flow model (Hu et al., 2014), we assessed the effect of pollen competitiveness on the gene flow frequency and quantified the threshold distance of gene flow, analyzed its spatial distribution characteristics, and made identified high‐risk and low‐risk regions in 24 provinces. These could provide scientific data for setting proper isolation distances between GM maize and non‐GM maize and identifying the optimal locations for hybrid maize seeds.

**TABLE 2:** Maize tasseling and flowering phase in 24 provinces, municipalities and autonomous in China

Location	Time of Tasseling and flowering phase
Anhui	Early August to late August
Chongqing	Late May to mid‐July
Guangdong	Late May to late September
Guangxi	Early May to mid‐September
Guizhou	Mid‐June to early October
Hainan	annual
Hebei	Early July to mid‐September
Heilongjiang, Jilin, Liaoning, Gansu	Mid‐July to mid‐August
Henan	Late July to late August
Hubei, Yunnan	Early June to mid‐August
Hunan	Late June to early July
Inner Mongolia, Shanxi	Mid‐July to early September
Jiangsu	Mid‐June to late August
Ningxia	Mid‐July to early August
Shandong	Late July to early September
Shanxi	Early July to early September
Sichuan	Early June to early September
Tianjin	Mid‐August to late August
Xinjiang	Mid‐July to late July

Note

The data were analyzed from the China Meteorological Data Service Centre (http://data.cma.cn).

## 2 MATERIALS AND METHODS

### 2.1 Maize gene flow model

The maize gene flow model used in this study was based on Gaussian plume model, which is appropriate for small‐scale pollen diffusion under the uniform surface and steady turbulent. This model used conventional meteorological data obtained from the China Meteorological Data Service Centre (including wind speed and direction, temperature, relative humidity, and sunshine duration) as input. Therefore, this model could be spread out over a larger area where there is no gene flow field experiment data and calculate the gene flow distance to provide scientific data for setting the measurement to control the transgenic flow.

### 2.2 Simulating the pollen diffusion

The Gaussian diffusion formula as follows was used to estimate the contribution of a continuous point source of pollen grains at the position (*i*, *j*, *z*_H_) to the pollen concentration at a downwind site (*x*, *y*, *z*), which was described detailed in our paper (Hu et al., 2014):

(1) $$C\left(x,y,z,i,j,z_{H},t\right)=\frac{Q(i,j,z_{H},t)}{2\pi {\bar{u}}_{H}\sigma_{y}\sigma_{z}}\exp\left[‐\frac{{\left(y‐j\right)}^{2}}{2\sigma_{y}^{2}}\right]\exp\left\{‐\frac{{\left[z‐z_{H}+v_{d}\left(x‐i\right)/{\bar{u}}_{H}\right]}^{2}}{2\sigma_{z}^{2}}\right\}$$

where *C* (grain m^−3^) is the pollen concentration in the air, *z*_H_ (m) is the height of the tassels, *v_d_* (m s^−1^) is the settling speed of pollen grains, and ${\bar{u}}_{H}$ (m s^−1^) is the wind speed at the tassel height. *σ*_y_ and *σ*_z_ (m) are the crosswind and vertical diffusion parameters, respectively, which represent the standard deviation of the pollen concentration distribution in the horizontal and vertical directions. *Q* (grain s^−1^) is the pollen source strength. Our experiments showed that each plant shed about 4.85–12.3 × 10^6^ pollen grains from anther; however, many of them were captured by canopy and deposited in situ or nearby and only a tiny portion could escape above the canopy and disperse to the downwind; here, the portion was empirically determined to be 15.82% in the field experiment. On the contrary, our model found, as the area of pollen source increased, the concentrations in the air also increased, but this effect leveled off with the larger source areas. The concentrations reached an inflection point and grew slower when the pollen source was larger than 100 × 100 m^2^. So, the donor area was set as 100 m length and 100 m width and the recipient was 1000 m length and 100 m width in this study.

In the process of maize pollen transmission within the canopy, many of pollen grains are intercepted by the upper leaves, and some of pollen grains penetrate into the canopy to reach the ears. Similar to light transmission, the pollen distribution in the canopy depends on the canopy structure, which is inversely proportional to leaf area index (Dietiker et al., 2011; Maddonni et al., 2001). The deposition of pollen grains from the donor is:

(2) $$D_{\text{donor}}=\exp\left(‐k_{p}\cdot L\right)\cdot C_{\text{donor}}\cdot v_{d}$$

While the deposition of pollen grains from the recipient is:

(3) $$D_{\text{recipient}}=\exp\left(‐k_{p}\cdot L\right)\cdot Q_{\text{recipient}}+\exp\left(‐k_{p}\cdot L\right)\cdot C_{\text{recipient}}\cdot v_{d}$$

where *L* is the cumulative leaf area index above the height of ears and *k*_p_ is the coefficient of pollen grains intercepted by the canopy; here *k*_p_ = 0.55, which was fitted by the experimental data. $\exp(‐k_{p}\cdot L)$ is the percentage of pollen grains through the canopy.

### 2.3 Simulating the gene flow frequency

The seed‐setting rate depends on the amount of pollen grains deposited on the filaments of ears. The more the pollen grains deposited on the filament, the higher the probability of fertilization. In theory, when no donor pollen in the pollen mixture, the gene flow frequency (*G*) is equal to 0%; and when no recipient in the pollen mixture, *G* is equal to 100%. Namely,

(4) $$\left\{\begin{array}{cc} G=0\%, & \frac{D_{\text{donor}}}{D_{\text{donor}}+D_{\text{recipient}}}=0\\ \;G=100\%, & \frac{D_{\text{donor}}}{D_{\text{donor}}+D_{\text{recipient}}}=1 \end{array}\right.$$

where *D*_donor_ and *D*_recipient_ are the number of pollen grains deposited on the filaments from donor and recipient, respectively. The ratio of $\frac{D_{\text{donor}}}{D_{\text{donor}}+D_{\text{recipient}}}$ represents the quantitative pollen competitiveness between the donor and recipient.

And beyond that, our study revealed that even if the same amount of pollen grains was pollinated on the filaments, the outcrossing rates were different for the different parental combinations, which was detailedly shown in “2.4 Parameterizing the genetic competitiveness” and “3.1 Genetic and quantitative competitiveness between the donor and recipient.” This demonstrates that not only pollen quantitative competitiveness, but also genetic competitiveness between the donor and the recipient also affects the gene flow frequency. So, a new parameter, *cp*, was introduced into the model to describe the genetic competitiveness, which is equal to the outcrossing rate when the donor pollen had equal amounts with the recipient pollen, namely,

(5) $$G=cp\times 100\%,\;\frac{D_{\text{donor}}}{D_{\text{donor}}+D_{\text{recipient}}}=0.5$$

Combined the observational data with the above‐mentioned conditions (4–5), the relationship between gene flow frequency and the pollen deposition can be expressed as follows:

(6) $$G=\left[A^{\frac{D_{\text{donor}}}{D_{\text{donor}}+D_{\text{recipient}}}}‐1\right]/\left(A‐1\right)\times 100\%$$

where *A* is an intermediate variable related to *cp*, $A={\left(\frac{1‐cp}{\mathrm{cp}}\right)}^{2}$, *cp* is the genetic competitiveness parameter, it ranges from 0 to 1 theoretically, and the practical upper limit is less than 0.5.

To simulate the worst‐case scenario, *cp* = 0.5 needs to be considered in the aspect of genetic competitiveness, on the other aspect of quantitative competitiveness, assuming the synchronization between the anthesis of the donor and the silking of the recipient, which led to higher gene flow frequency compared with the asynchronized case.

### 2.4 Parameterizing the genetic competitiveness

Genetic competitiveness is described as a parameter of the outcrossing rate when two or more kinds of pollen grains are pollinated on the same filament (Rognli et al., 2000). To determine the pollen genetic competitiveness, we conducted an experiment on the artificial pollination of pollen mixture from multi‐varieties (Hu et al., 2014). We used Zinuo18 (Pz), Jidan35 (Yj) to conduct the artificial pollination experiments. The endosperm of “Pz” is white and the seed coats of “Yj” are yellow, which are the recessive homozygous traits, the endosperms of “Yj” are yellow and the seed coat of “Pz” is purple, which are the dominant homozygous traits. Pollen grains of “Pz” and “Ys” were weighted to made 15 combinations of pollen mixtures, including “Pz” pollen: “Ys” pollen as 0:100, 1:99, 5:95, 10:90, 20:80, 30:70, 40:60, 50:50, 60:40, 70:30, 80:20, 90:10, 95:5, 99:1, and 100:0. These mixtures were artificially pollinated to the filaments of Pz and Yj, namely (Pz + Yj) × Pz and (Pz + Yj) × Yj. After maturation, the color of the endosperm or seed coat was used to assess the selfing and/or outcrossing of the ears. When the pollen grains from two varieties were equal in quantitative terms, the ratio that the grains produced by outcrossing divide by the total grains on the ears was considered as the parameter of genetic competitiveness.

### 2.5 Geographical area and time scope

The input data all came from the China Meteorological Data Service Centre (http://data.cma.cn). As Table 2 shows, the maize flowering phase in 24 provinces, cities, and autonomous was determined by the crop growth and development dataset (1992–2014). Sixty years (1951–2015) of surface meteorological data in these regions were used as an input, from which we calculated the gene flow frequency of each distance in 2057 maize‐producing counties.

Hainan Province is not a major producer of maize, but it has sunny and hot climate. A large number of maize breeding materials are generated and propagated here every year, and it has become an indispensable way to shorten the breeding period and solve the shortage of stock seeds and breeder seeds. The Sanya City, Lingshui, and Ledong County in southern Hainan have developed into a major area or center for Hainan crop winter‐season multiplication (HCWM), including agricultural GMOs. Significant challenges include ensuring the purity of the varieties being tested, avoiding or reducing contamination between these seeds, and preventing the gene flow from GM materials. Therefore, this study will analyze the gene flow distance of the southern area separately during the relatively scattered flowering phases.

### 2.6 Thresholds of gene flow

The maize gene flow model used a negative exponential function to simulate the gene flow frequency. This required establishing a threshold; otherwise, the gene flow distance could be infinite. China's National Standard for Maize Seed Quality (GB 4404.1–2008) outlined the seed purity thresholds as follows: for conventional maize, stock seed ≥99.9% and production seed ≥97.0%; for inbred lines: stock seeds ≥99.9% and production seeds ≥99.0%; for single‐cross seeds ≥96.0%; for double‐ and triple‐cross seeds ≥95.0% (MARA, 2008). In this study, we confirmed 1% and 0.1% as the threshold values, which met the purity requirements of production seed and breeder seed.

The current labeling systems for GM agricultural products are as follows: EU’s labeling threshold is 0.9%, Taiwan's threshold is 3%, Brazil's threshold is 1%, Korea where the threshold is 3% and Japan, Hong Kong, and the United States is labeling thresholds of 5% (Beckie & Hall, 2008). This means that if GM ingredients constitute more than 0.9%, 1%, 3%, or 5% of the final product, the GM materials must be disclosed on the label. After considering both safety and cost, the thresholds of 1% and 0.1% were selected for isolation distance. These thresholds not only meet seed quality purity requirements, but comply with international trade regulations.

### 2.7 Maximum threshold distance (MTD)

The 1% and 0.1% threshold distances (TD_1_% and TD_0.1_%) refer to the distance when the gene flow frequency is less than or equal to 1% and 0.1%, respectively. TD_0.1_% means that, out of 1000 plants, there will be one or fewer plants generated by gene flow outside the threshold distance. In this case, the seed purity reaches ≥99.9%. In this study, the thresholds were ruled as 1%; that is, the GM ingredients in agricultural products outside the threshold distance will be lower than most of the above labeling thresholds.

Specific steps are as follows: first, the TDs of different years were estimated by using the maize gene flow model, with wind speed, wind direction, air temperature, relative humidity, and sunshine duration as input; second, the maximum threshold distance (MTD_1%_ and MTD_0.1%_) of a certain place could be found from the annual TDs, which provided the worst‐case scenario of maize gene flow risk for a certain period. Here, 65 years of meteorological data (1951 to 2015) were applied to analyze and improve the reliability and stability of MTDs.

## 3 RESULTS

### 3.1 Genetic and quantitative competitiveness between the donor and recipient

**FIGURE 1:**
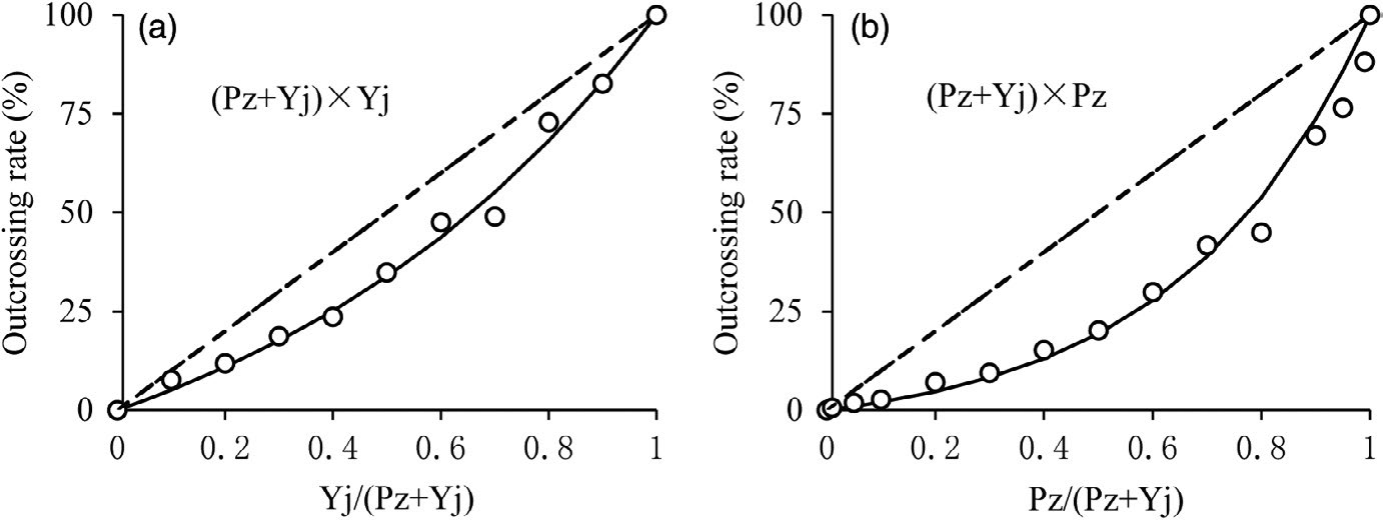
Outcrossing rate under the different combinations of pollen mixture. Pz and Yj represented the maize materials, Zinuo18 and Jidan35, respectively. (Pz + Yj) × Yj indicated that the pollen mixture made by Pz and Yj was pollinated on the filament of Yj, while (Pz + Yj) × Pz indicated this mixture was pollinated on the filament of Pz. Pz/(Pz + Yj) represented the percentage of Pz in the pollen mixture of Pz and Yj, while Yj/(Pz + Yj) represented the percentage of Yj in this mixture

The artificial pollination of pollen mixtures showed that the outcrossing rate was always increasing with the increase in alien pollens in mixture. It is worth noting, however, that when the amount of alien pollens was equal to that of native pollens in the mixture, the outcrossing rates were a far cry from the different combinations, as Figure 1 shows. The former represented the quantitative competitiveness between the two species of pollen grains, and the latter represented the genetic competitiveness.

The experiments quantified the genetic competitiveness as 0.348 for (Pz + Yj) × Yj and 0.202 for (Pz + Yj) × Pz. They were a relative value of one species outcrossing rate versus another. The higher the genetic competitiveness, the higher the possibility that the donor's pollens were outcrossed with the filaments of recipient, and the greater the gene flow frequency and the threshold distance. In this study, the genetic competitiveness was always lower than 0.5; it means that the native pollens were more likely to fertilize on the filaments of ears than the alien pollens.

To make the model applicable to different maize varieties, three types of genetic competitiveness were set to assess the threshold distances: *cp* = 0.05, *cp* = 0.25, and *cp* = 0.5. When *cp* = 0.5, the genetic competitiveness between the donor and recipient was equal, when *cp* = 0.05, the pollen competitiveness of donor was much lower than the recipient, and *cp* = 0.25 is when the competitiveness was intermediate.

### 3.2 Statistics of the maximum threshold distances of maize gene flow in China

**FIGURE 2:**
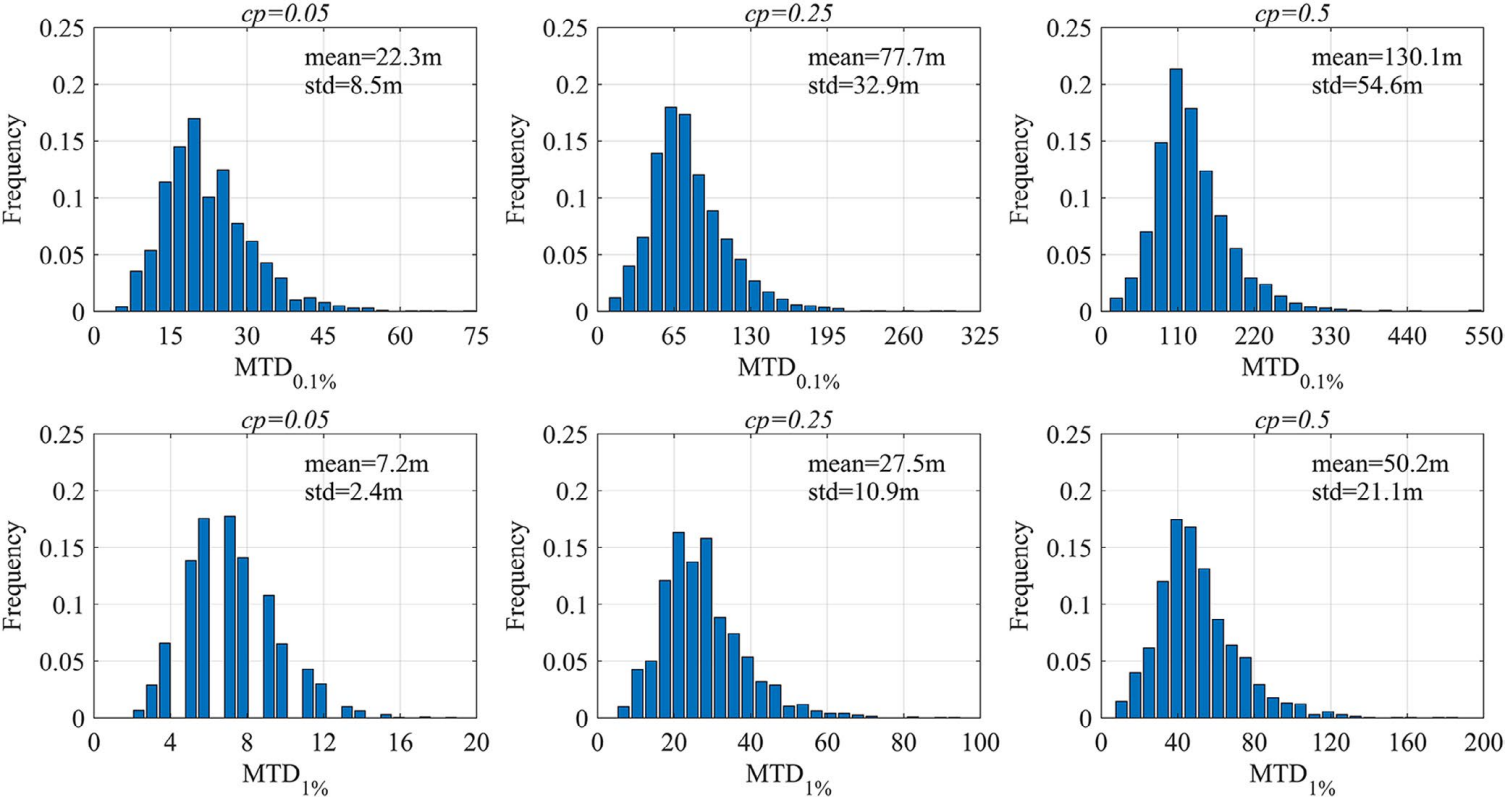
Frequency distribution of the maximum threshold distances (MTD) for maize gene flow in China. MTD_1%_ represents the MTDs at 1% threshold, and MTD_0.1%_ represents the MTDs at 0.1% threshold

As Figure 2 shows, a large difference in MTD was considerable across China due to the different climates, with coefficients variation as high as 32.6%–42.4%. Under the same genetic competitiveness and threshold conditions, the maximum MTD value was 19.0–58.4 times the minimum value, making it necessary to analyze the spatial distribution of the maximum threshold distance of maize gene flow in China.

The gene flow distances in different sites were approximately normally distributed, with the MTDs in most sites concentrated near the average value. Only 0.5%–2.7% of the sites exceeded the average value by more than 2 times. While the national maximum MTD was 548 m (far greater than GM maize's reference isolation distance of 300 m), at a genetic competitiveness of 0.5 and a threshold value of 0.1%, more than 95% of areas had MTD_0.1%_ concentrated within 302 m and less than 0.2% of areas had an MTD exceeding 500 m.

Genetic competitiveness and threshold value were important factors that affect MTDs. When the threshold value increased by one order of magnitude, from 1% to 0.1%, the average MTD increased 1.6–2.1 times. During the genetic competitiveness increased from 0.25% to 0.5%, the average MTD increased by 0.7–0.8 times. As genetic competitiveness decreased, MTD changed more significantly; average MTD differed by 2.5–2.8 times after the genetic competitiveness decreased from 0.25 to 0.05. However, under different threshold values and genetic competitiveness, the spatial distribution pattern of MTDs was changed a little bit and the correlation coefficient between MTDs reached above 0.9.

### 3.3 Spatial distribution of the maximum threshold distances in China

**FIGURE 3:**
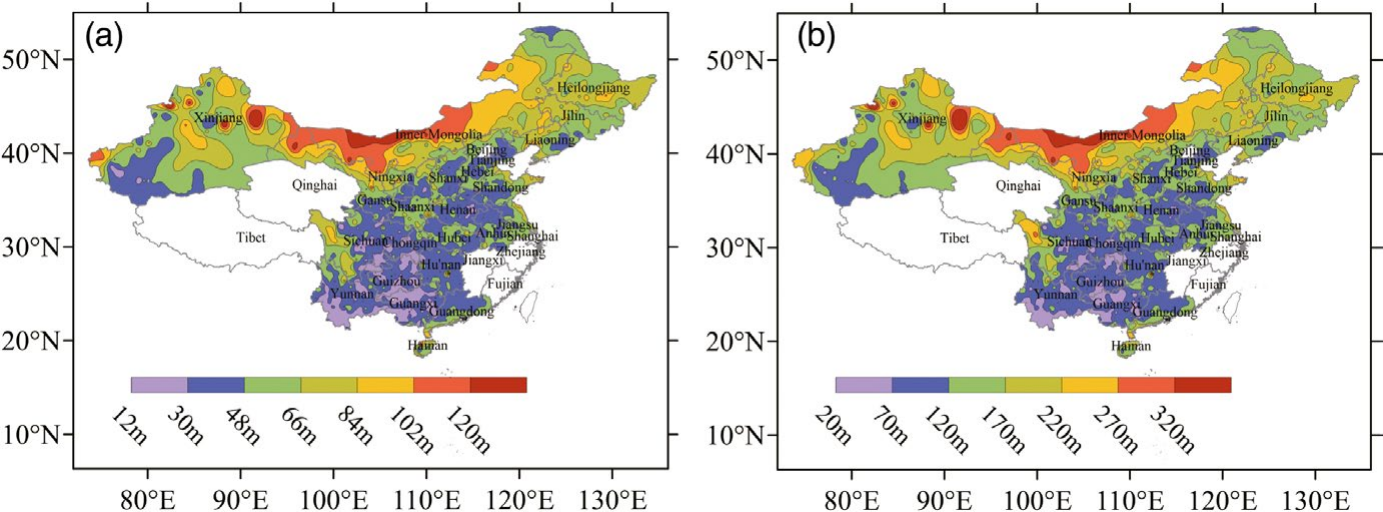
Spatial distribution map of the maximum threshold distance of maize gene flow in China at 0.5 of the genetic competitiveness parameter. (a) represents the MTDs at 1% threshold, and (b) represents the MTDs at 0.1% threshold. The blank represents the regions with fewer maize planting

Here, the case of *cp* = 0.5 was used to assess the spatial distribution of MTDs and the contour map of MTDs in 24 provinces of China was drawn as Figure 3. It was shown that the MTDs gradually decrease from north to south and from east to west. According to the MTDs, the risk of China's maize gene flow could be divided into three levels, high‐risk regions, low‐risk regions, and medium‐risk regions.

There was a long and big belt with high risk of gene flow in the north of China that covered most of Inner Mongolia and the western regions of Heilongjiang, Jilin, and Liaoning and extended to the northern regions of Gansu and Xinjiang. Among these provinces, Inner Mongolia and Heilongjiang had the highest MTDs. The coastal areas of Liaoning, Shandong, Guangdong, and Hainan also had a long and narrow high‐risk gene flow zone. The other high‐risk regions were located at the south of Songnen Plain and north of Liaohe Plain, where maize production was concentrated, known as China's “maize‐planting belt.” In addition, it could not be ignored that these regions had a significant risk for maize gene flow.

The low risk of gene flow was primarily concentrated in southwest China. Chongqing, Guizhou, Yunnan, and Guangxi were the four provinces with the shortest gene flow distances. The Eastern Henan Plain had a relatively lower risk of gene flow, too. However, Sichuan Province had a special distribution pattern, and its eastern region, the Sichuan Basin, together with Yunnan–Guizhou Plateau, and Guangxi Basin, were the largest low‐risk areas for gene flow, while its western region, the Hengduan Mountains, was the high‐risk areas for gene flow.

The following was further explored the detailed sites with high or low MTDs to seek the suitable GM maize plantation and control the transgenic flow. As such, we sorted these regions according to their MTD values and selected the highest 5%, MTD_0.1%_>228 m or MTD_1%_>89 m, as the high‐risk sites and the lowest 5%, MTD_0.1%_<56 m or MTD_1%_<21 m, as the low‐risk sites.

**FIGURE 4:**
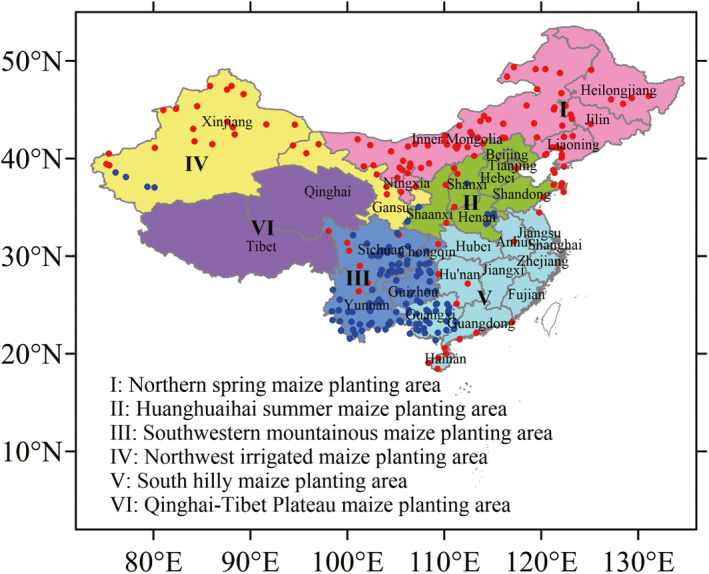
High‐ and low‐risk sites of the maximum threshold distance of maize gene flow in China. Blue circles represent low‐risk sites, and red circles represent the high‐risk sites

Figure 4 shows the distribution of high‐ and low‐risk sites. The most high‐risk sites were intensively distributed over the northern spring maize area and the northwest irrigated maize area. Of them, Inner Mongolia has the most high‐risk site accounting for 37% of all high‐risk sites, and 40.3% of all sites in the province was the high‐risk sites, followed by Xinjiang, of which Alashankou and Qijiaojing are the two sites with largest MTD_0.1%_ and MTD_1%_. The Huanghuaihai summer maize area and the southern hilly maize area have long coastlines, and there were many high‐risk sites in these regions; particularly in Hainan Island, 21.1% sites were high risk at risk of gene flow. The most low‐risk sites were distributed mainly in the southwest mountainous maize area. The four provinces of Guangxi, Yunnan, Guizhou, and Chongqing in the southwest had more than 70% of the low‐risk sites. The correlation coefficient was shown as 0.87–0.89 (n = 52) between the wind speeds and MTDs at high and low sites. It was the wind speed that caused the spatial heterogeneity of MTDs.

### 3.4 The maximum threshold distances in the Hainan Crop Winter‐season Multiplication (HCWM) area

The Hainan Crop winter‐season multiplication (HCWM) area, including Sanya City, Lingshui, and Ledong County, is the main region where a lot of breeding organizations in our country engaged on the crop breed and propagate in winter. This place is occupied less than 6000 km^2^ of land area, accounting for 1.4% of China's maize‐planting area (NBS, 2020), but the variation range of MTDs could reach 127 m (MTD_0.1%_) and 46 m (MTD_1%_) with variable coefficients of 44% and 41%, respectively. It is of great significance to select the areas with lower risk of gene flow for breeding in order to avoid or reduce the interpollination between the breeding varieties and ensure their purity.

**FIGURE 5:**
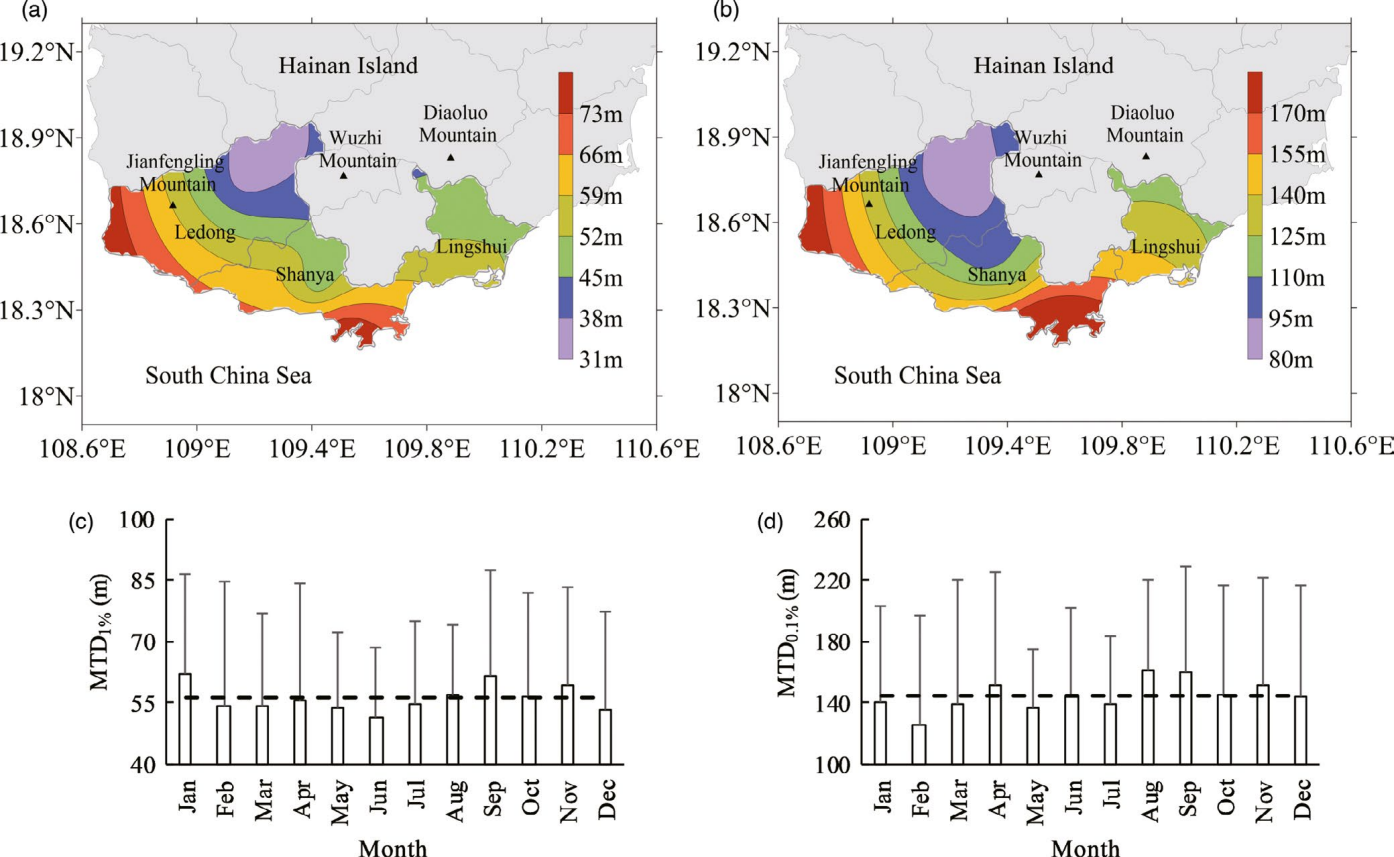
Maximum threshold distance (MTDs) in the Hainan crop winter‐season multiplication (HCWM) area. (a) and (b) are the spatial distribution of annual average MTDs, and (c) and (d) are monthly change of average MTDs in HCWM. (a) and (c) represent the MTDs at 1% threshold, and (b) and (d) represent the MTDs at 0.1% threshold

Figure 5a and b displays the spatial variation of MTD_1%_ and MTD_0.1%_ in the HCWM area, using *cp* = 0.5 as an example. Our results demonstrated that there were two high‐risk gene flow areas in this region, which were located in the coastal areas of southern Sanya City and western Ledong County. Gene flow distances gradually decreased from the coast to inland and from south to north. The risk of gene flow in Sanya City is the highest, with an annual average MTD_0.1%_ and MTD_1%_ of 213 and 80 m, respectively.

The HCWM area was subject to monsoons, where the northeast monsoon prevailed in winter, while spring and early summer were mostly affected by the southeast monsoon from the South Pacific and southwest monsoon from the Indian Ocean. According to the wind frequency statistics, the frequency of north‐northeast winds in these three regions was similar in winter (34.3% in Sanya City, 35.2% in Lingshui County, and 38.6% in Ledong County). The frequency of wind direction differed during the spring and summer that the frequency of south‐southeast winds was highest in Lingshui County (40.2%), while the lowest was in Ledong County (11.3%). The frequency of west‐southwest winds was the opposite in these regions, Ledong County had the highest (15.2%), and Lingshui County was the lowest (6.8%). It followed that Lingshui County was greatly affected by southeast monsoons, while Ledong County was more affected by the southwest monsoons.

The HCWM area was surrounded by the sea on three sides, with Lingshui County to its east, Ledong County to its west, and Sanya City in the middle. Northeast Ledong County, northern Sanya City, and northwest Lingshui County were all parts of the Wuzhi Mountain System. The main peak of Wuzhi Mountain was 1867 m above sea level in the north of Sanya, and Jianfengling Mountain, in the northeastern mountainous area of Ledong County, was approximately 1000 m above sea level, and the Diaoluo Mountain, in northwest Lingshui County, was approximately 1519 m above sea level. They were a large topographic barrier, which impact on the wind speeds in the HCWM area and significantly weaken the wind speed near Wuzhi Mountain. The two high‐risk areas were both located in the coastal plains of the HCWM area, where the wind speeds were higher and there were not the topographical barriers to protect by and directly impact by the monsoons. It was the reason for the MTDs pattern in the HCWM area.

Maize could be grown all the year round in HCWM area, though the flowering periods differed due to the different sowing times. Comparison with monthly MTDs could help select the months with shorter MTDs and reduce the gene flow rate and risk. Figure 5c and d displays the annual variation of MTDs in HCWM base, using *cp* = 0.5 as an example. The results showed that MTD_0.1%_ was shortest in February and longest in August, while MTD_1%_ was shortest in June and longest in September. However, there was no obvious periodicity for monthly MTDs. With some planning, we could be in position to take advantage of the low‐risk period which helps meet that need. February and March were the months when the gene flow risk was relatively small.

## 4 DISCUSSION

Maize is one of the most important food crops and the second‐largest GM crop in the world. Mainly, maize was distributed in the United States (31.3%), China (23.5%), Basil (9.1%), and the European Union (5.8%), coupled with Mexico, the center of origin of maize, where a lot of gene flow experiments were conducted (USDA, 2020). To explore the law of transgenic flow in maize, they used morphological markers, glyphosate‐resistant or insect‐resistant maize as pollen donors and collected nontransgenic maize samples at various distances around transgenic maize where GM maize and non‐GM maize coexist. These experiments were scattered over 13 countries, which covered the major maize production in the world. A total of 44% of them were concentrated in EU countries; the highest number of experiments were carried out in the Spain and United States. China also conducted many experiments to assess the possible risks of gene flow in maize prior to commercialization of its transgenic type. The results found that a large difference existed in the MTD_1%_ and the MTD_0.1%_, as shown in Table 1. The median value was 32 m and 116 m, respectively; however, the maximum value exceeded 110 m for MTD_1%_ and reached 780 m for the MTD_0.1%_, which complicated the isolation distance managements.

To provide an objective basis for setting isolation distances for GM maize as well as spatial isolation for the inbred lines reproduction and hybrid maize seeds production, this study used a maize gene flow model to calculate the maximum threshold distance for the gene flow frequency equal to or lower than 1% and 0.1%. This method was put into practice at 2057 maize‐growing counties of China, and the results showed that the MTD_1%_ and MTD_0.1%_ were 2–187 m and 4–548 m, respectively. In contrast with the field experiments, 95% of our MTD_1%_ value and 65% of our MTD_0.1%_ value were within the range of the field experiments in Table 1; the correlation coefficient between the modeling results and the field data is 0.663 in the five experiments of Heilongjiang, Shandong, Jilin, and Hainan in China, which confirmed that our results were credible. It not only provided scientific data to set the isolation distance for China's GM maize, but also referenced in other countries.

It was deficiency that the uncertainty of the results was increasing, as the threshold became more restrictive, which was very similar to the results from field experiments. This study relied on the maize gene flow model to simulate and predict the MTDs, which was limited by the spatial and temporal resolutions of meteorological data and the accuracy of the maize gene flow model. Wind speed had a large spatial heterogeneity, particularly under complex terrain conditions, which increased the uncertainty of the MTDs. Therefore, it was necessary to perform systematic and long‐term ecological environmental monitoring following the commercialization of GM maize, including investigating the distribution of different GM maize varieties and the environment of GM maize‐growing areas, to prevent the potential risks.

We also introduced a new variable, the genetic competitiveness parameter, into the maize gene flow model. The genetic competitiveness parameter describes the preference when GM pollen and non‐GM pollen both fall on the same filaments of nontransgenic maize, which is known as hybrid compatibility. In previous maize gene flow models, it was thought that the receptor had no preference for pollen grains from different varieties and the probability that the pollen grains of different varieties fertilized different filaments was equal (Angevin et al., 2008; Arritt et al., 2007; Aylor et al., 2003; Coléno et al., 2009; Dietiker et al., 2011; Jarosz et al., 2004; Lipsius et al., 2006; Loos et al., 2003). However, maize possessed the hybrid incompatibility, a genetic trait that does not change with the environmental conditions. This incompatibility was controlled by the *GA* or *GA* alleles of the dominant gametophyte gene (Zhang et al., 2018). Maize with the dominant *GA* gene could fertilize with other varieties, while the pollen of other maize could not unless it possessed the same dominant gene. In the genus maize, there were complete hybrid and varying degrees of partial incompatibility. This meant there would be three different conditions in the maize hybrid experiment. It was difficult for the varieties with a recessive *GA* gene to be pollinated by a plant with a dominant *GA* gene, while the dominant homozygous *GA* gene pollen could be pollinated by a dominant pure *GA* gene. The hybridization rate of pollen from the *GA* gene varieties containing the dominant heterozygous *GA* gene was between the two scenarios mentioned above (Kermicle & Evans, 2010; Lu, et al., 2020). In most cases, the pollen from the corn itself was easier to fertilize on the filaments than the foreign pollens. Theoretically, the same amount of mixed pollen from two different varieties was pollinated on the filaments of one variety; the outcrossing rate could not exceed 50%. Therefore, we designed three different genetic competitiveness parameters: *cp* = 0.05, *cp* = 0.25, and *cp* = 0.5, representing hybrid incompatibility, partial compatibility, and complete compatibility, respectively.

The quantitative pollen competition is the dominating factor in field conditions, comparing with the genetic competitiveness. A single plant produces about several million pollen (Fonseca et al., 2004; Uribelarrea et al., 2002); however, the portion of pollen grains escaped and dispersed downwind is very little, which was only 15.82% in our study, and fewer pollen grains could penetrate into the canopy and reach the ears, which was the basic of gene flow (Hu et al., 2014). The weighting of transgenic pollen grains in the pollen mixture on the filaments, which was called quantitative pollen competition in our study, plays a leading role in determining the transgenic flow risk when GM maize coexisted with and non‐GM maize. It showed that the bigger the weighting, the more likely the pollen grains were to fertilize on the filaments. The flower synchronization between the donor and recipient was a decisive factor to effect this weighting. The anthesis‐silking interval (ASI) in maize was another contributing factor. ASI in non‐GM maize may be a very crucial factor as delay of silking will reduce the quantitative pollen competition itself and provide more possibilities to cross with foreign GM pollen, especially with climate change, the abiotic stress is increasing, such as drought and high temperature, which is causing that ASI outbreaks have been widespread in major maize‐producing areas (Liu et al., 2021). When the delaying silking of non‐GM maize just met with the anthesis of GM maize, ASI would increase transgenic flow risk due to lack of the pollen competition from nontransgenic plant.

Reasonable isolation measures can effectively prevent and control the ecological and economic risks caused by gene flow. Our results demonstrated that the MTD_1%_ and MTD_0.1%_ between the complete compatible varieties were within 548 and 187 m, respectively. The current reference isolation distance of GM maize is 300 m in China, which can reduce the gene flow frequency to less than 1% and meet the requirements of most countries and regions for non‐GM products. In China, an isolation distance of 300–500 m is typically used in maize seed production (Xing et al., 2006). An isolation distance of 300 m achieves a seed purity of 1%, which is suitable for producing hybrid and production seeds. Except for some individual sites (Alashankou and Bajiaojing in Xinjiang), more than 99.9% of MTD_0.1%_ are within 500 m. Therefore, for most areas, the isolation distance of 500 m can yield a seed purity of better than 0.1% and meet the production needs of breeder seeds. If GM material contained a *GA* gene, its risk of gene flow would be greatly reduced and the MTD_1%_ and MTD_0.1%_ between hybrid incompatible varieties would be within 19 and 75 m, respectively, unless the adjacent non‐GM maize also contains a GA gene. The isolation distance of 300 m can keep the gene flow frequency below 0.1%, which meets the production requirements of maize breeder seeds.

On Jan 21, 2021, the Ministry of Agriculture and Rural Affairs issued three “Agricultural GMO Safety Certificates” for insect‐resistant and herbicide‐tolerant GM maize, approving the GM maize DBN9936 for nationwide use in different ecological zones, including the northern spring‐planting area, the Huanghuaihai summer‐planting area, the southwest mountain area, the southern hilly area, and the northwest irrigated area (MARA, 2021). The commercial planting of GM maize is an inevitable trend, but at the initial stage, there is an unavoidable risk of exogenous gene flow, when GM maize and non‐GM maize are in coexistence. In order to guarantee the development of maize industry and promote the healthy industrialization of transgenic maize, it is necessary to refine the scientific and reasonable threshold management measures and optimize and adjust the transgenic flow control measures.

In China, 85% of maize is grown in a narrow strip from the northeast to the southwest through north China, which runs over the northern spring area, the Huanghuaihai maize area, and the southwestern maize area (NBS, 2020). Our research demonstrated that the MTD_1%_ and MTD_0.1%_ of the northern spring maize were approximately 40%–43% higher than the Huanghuaihai maize area and 168%–173% higher than the southwest maize area. The northwest irrigated maize area is currently the primary region producing hybrid of maize in China, while Gansu and Xinjiang are the two largest hybrid seed production provinces in China. Zhangye City in Gansu Province is the capital of maize hybrid seed production, accounting for approximately 25% of China's output, and Changji Prefecture has the highest concentration of maize hybrid seed production in Xinjiang, accounting for approximately 15% of China's output (NBS, 2020). However, there is a high risk of GM maize gene flow in these places. The MTD_1%_ in Zhangye is 71 m and in Changji is 69 m, while the MTD_0.1%_ in Zhangye is 195 m in Changji is 155 m. If adjacent maize was incompatible, the risk of gene flow would be greatly reduced.

The region of Sanya City, Lingshui, and Ledong County in the southern Hainan are three of the highest‐risk areas for gene flow, which is the winter breeding center for many crop materials all over the country. If GM maize contaminates the HCWM area, the exogenous gene can easily be brought to the mainland with the southern breeding seeds. This is an undesirable outcome for controlling the gene flow of GM maize. Therefore, it is extremely important to identify regions and times that pose the lowest risk of gene flow during the southern breeding. Due to the southern breeding area with the small size and the short season, it is difficult to implement time and space isolation measures to prevent cross‐contamination (Zhang et al., 2020). As such, the promotion and application of GM maize must be treated cautiously in the southern breeding area. This study found that the spatial heterogeneity of MTDs is still large, even at the areas less than 6000 km^2^ in the HCWM area. In the coastal plain of southern Sanya City, the MTD_0.1%_ is over 200 m, while the MTD_0.1%_ of the region around Wuzhi Mountain is less than 100 m. Alternatively, to avoid August to October of typhoon season in the HCWM area and ensure that the maize was harvested, dried, and packed before the rainy season of April in the following year, the maize bred in the southern generally sowed from late October to late November (Xing et al., 2006) and flowered just late March to keep away from the seasons with high MTDs. It is helpful to control the contamination risk and reduce the outcross between the breeding materials and ensure the purity of these varieties.

## CONFLICT OF INTEREST

The authors have no conflicts of interest to disclose.
